# Fulminant Hepatic Failure in Glucose-6-Phosphate Dehydrogenase (G6PD) Deficient Patients Caused by Hepatitis E Infection: A Single Disease With Different Spectrums

**DOI:** 10.7759/cureus.12276

**Published:** 2020-12-25

**Authors:** Lubna Kamani, Hafeezullah Shaikh, Ajit Kumar Khemchandani

**Affiliations:** 1 Gastroenterology, Liaquat National Hospital, Karachi, PAK; 2 Gastroenterology and Hepatology, National Institute of Liver & GI Diseases (NILGID) Dow University Hospital (DUH), Karachi, PAK; 3 Gastroenterology, National Institute of Liver & GI Diseases (NILGID) Dow University of Health Sciences, Karachi, PAK

**Keywords:** hemolysis, fulminant hepatic failure, glucose-6-phosphate dehydrogenase (g6pd), acute hepatitis e

## Abstract

One of the major causes of acute viral hepatitis in Pakistan is the hepatitis E virus. Virus-induced liver inflammation within sight of glucose-6-phosphate dehydrogenase (G6PD) insufficiency might be related with intricacies, for example, extremely low hemoglobin, red blood cell (RBC) destruction, renal function collapse, a decline in brain function due to severe liver disease and even demise. Despite the two diseases being widespread, their effect on understanding patient ailment has not been studied in depth. Hemolytic anemia occurs as a complication of acute hepatitis. Nevertheless, the occurrence can ascend to a large percentage of patients harboring glucose-6-phosphate dehydrogenase (G6PD) insufficiency. Although being frequent in endemic countries, there is a lack of literature in understanding the synergistic effect of hepatitis E disease and G6PD inadequacy leading to fulminant hepatic failure and increased mortality in the absence of a liver transplant facility. Here we report a case of two brothers, both having G6PD deficiency. A 19-year-old male, the elder of the two brothers, came with three days of complaints of persistent vomiting and deep jaundice. On investigation, he was found to have acute hepatitis E. During his hospital stay, he became drowsy, comatose, and subsequently expired. The second patient, his younger brother, was a 15-year-old male who presented with similar history about one week after his demise. He was also managed conservatively and was subsequently discharged from the hospital.

## Introduction

More than 400 million people worldwide are recognized to have inherited inadequate glucose-6-phosphate dehydrogenase (G6PD) enzyme, making it the most widely recognized acquired catalyst inadequacy [1]. The frequency of G6PD insufficiency in Pakistan is about 1.8% [2]. Hemolytic anemia occurs in 23% of victims as the intricacy of acute hepatitis. Nevertheless, the occurrence can ascend to 70% in patients harboring G6PD insufficiency [3]. Fulminant hepatic failure includes proof of coagulation variation from the norm, generally an international normalized ratio (INR)≥1.5, and any level of mental transformation in a patient without prior cirrhosis and with a sickness of <26 weeks [4]. Despite the two diseases being widespread, understanding patients' ailments have not been accounted for and limited only to case reports [5-7]. 

G6PD inadequacy is an X-connected recessive inherited illness described by unusually low degrees of glucose-6-phosphate dehydrogenase (curtailed G6PD or 6PDH), an assimilation catalyst associated with the hexose monophosphate (HMP) shunt pathway, particularly significant in red blood cells (RBC) catabolism [8]. It is frequently considered and revealed as more common in males; nonetheless, heterozygous females are a more frequent genotype [9]. More than 400 diverse hereditary variations of G6PD deficiency have been counted; for 186 of them, the exact mutation is known: most are single base changes, leading to amino acid substitutions causing missense mutation [10]. Massive intravascular hemolysis with renal insufficiency, hepatic encephalopathy, and even demise has recently been noted [11]. This case report presents two patients with similar clinical manifestations and the same diagnosis but totally different outcomes. It leads to the need to investigate and identify other factors predicting outcomes in (G6PD) deficient patients infected by acute hepatitis E.

## Case presentation

Case 1

A 19-year-old male with no known co-morbidities admitted to the emergency department with complaints of persistent vomiting and yellowish discoloration of sclera for three days. On general assessment, he was profoundly icteric. He denied any history of alcohol or over the counter drug intake. The abdominal assessment revealed a normal examination with no visceromegaly, and the rest of the examination was unremarkable.

Lab examinations revealed hemoglobin (Hb) concentration of 13 mg/dl on the day of admission, white blood count (WBC) of 12900 cells/mm^3^ on admission, serum total bilirubin of 27.2mg/dl, serum glutamate pyruvate transaminase (SGPT) was 6400u/l, and prothrombin time (PT) was 24 seconds (control 9 seconds). Hepatitis A virus immunoglobulin M (HAV IgM), hepatitis B core antibody (anti-HBc) total, hepatitis B surface antigen (HBsAg), and anti-hepatitis C virus (anti-HCV) were all negative, while hepatitis E virus (HEV) IgM came out to be reactive. The final diagnosis of severe hepatitis E was made, and the patient was managed accordingly.

In the days that followed, total bilirubin heightened to 65.9mg/dl (direct fraction: 40.61mg/dl), and alanine aminotransferase (ALT) was 3400u/l. The hemoglobin (Hb) declined to 7.4 g/dl. PT rose to 31 seconds. The peripheral film showed macrocytes with spherocytes and rouleaux formation. Urine was reactive for hemoglobin. The plasma lactate dehydrogenase (LDH) level was 5700 U/L (range: 200 to 500 U/L), and plasma haptoglobin was imperceptible. Retic count was 1.4. Direct anti-globulin test (DAT) and indirect anti-globulin test (IAT) were negative. The microscopic examination of blood films and antigen-based rapid diagnostic tests (RDT) for malaria were negative. Wilson’s disease was excluded by normal ceruloplasmin levels and the absence of deposits of copper in a ring around the cornea on slit-lamp examination. The serum G6PD levels were less than 10% from normal values. The patient was supervised modestly, inclusive of evading all hepatotoxins and ensuring sufficient urine output, but with progressively deteriorating Glasgow Coma Scale score, the patient was later intubated.

Later, the patient suddenly desaturated, and at the same time, he developed hematuria. He had been given fresh frozen plasma and blood transfused about two units of pack cell volume, but he was continuously deteriorating, dropping hemoglobin, and despite all aggressive measures, he ultimately expired.

Case 2

A 15-year-old male, the younger brother of the above-mentioned patient, recently diagnosed as anti-HEV IgM positive with G6PD deficiency admitted to the emergency department with complaints of yellowish discoloration of sclera, persistent vomiting for two days, and drowsiness for one day. There was no history of alcohol or drug intake before coming to the hospital. On assessment, he was mildly icteric, abdominal assessment uncovered a non-tender abdomen with no visceromegaly, and the rest of the systemic examination was unremarkable. Lab examinations showed a hemoglobin concentration of 11.6gm/dl, WBC counts of 16300 cells/mm^3^. PT of 13 sec (control 9 sec). Total bilirubin concentration was 6.95gm/dl (conjugated fraction 5.56mg/dl), alanine aminotransferase of 1374 u/l. Serum amylase was 65. The urine** **detailed report was normal.Blood culture and sensitivity were negative. The ultrasound abdomen was normal. The patient was managed conservatively in the intensive care unit. He got better the next day; his total bilirubin decreased from the previous value to 5.4gm/l, prothrombin time was 11 s. Therefore he was shifted to the ward. On the third day, he was discharged home.

## Discussion

Meagre hemolysis related to diminished red blood cell viability might be routinely observed with virus-induced liver inflammation yet is rarely a clinical consequence [12, 13]. Even so, when virus-induced liver inflammation happens in G6PD-insufficient patients, hemolysis might be serious [13, 14]. In our case report, the first patient had serious hemolysis occurring in the vasculature as proven by a fall in hemoglobin, raised bilirubin in the blood, hemoglobin excretion in urine, imperceptible plasma haptoglobin degrees, and heightened serum lactate dehydrogenase level. The existence of extremely raised serum bilirubin levels in patients with viral-induced liver inflammation and G6PD insufficiency has been recently revealed [15-18]. The effects of G6PD insufficiency on cases with hepatitis A infection was evaluated by Gotsman and Muszkat in a case-control study [19]. Significant hemolysis in G6PD-lacking people is normally accelerated by the introduction of exclusive drugs. Nonetheless, as for this situation, virus-induced liver inflammation might hasten substantial hemolysis even without the admission of such medications [13, 18]. When G6PD lack is alleged, therapy with drugs causing hemolysis ought to be evaded in light of the fact that it might also exacerbate hemolysis [20].

The system of hemolysis is anticipated to happen through diminished degrees of reduced glutathione in erythrocytes. [12]. Declined glutathione levels could transpire from the amassing of oxidative agents because of hepatic ailment and give rise to expanded hemolysis within sight of G6PD lack. Despite the significant bilirubin levels in these cases, the foreboding is mostly identified with the seriousness of liver injury [15].

HEV disease is communicated through the feco-oral course. At the same time, in contrast to other enteric operators, it doesn't commonly spread from contaminated people to their nearby contacts.

In the above two cases, both brothers acquired HEV infection, and both had G6PD deficiency. The elder brother developed fulminant hepatic failure, hemolysis and ultimately died. While the younger sibling did not develop these complications, he became better and discharged home.

## Conclusions

In patients with severe virus-induced liver inflammation and unexplained low hemoglobin levels with heightened bilirubin concentrations, hemolysis in vasculature should be thought of and researched as it could be a prognostic factor in these patients.
